# The genome sequence of the Common Sun Beetle, *Amara aenea* (De Geer, 1774) (Coleoptera: Carabidae)

**DOI:** 10.12688/wellcomeopenres.28085.1

**Published:** 2026-10-09

**Authors:** Mark G. Telfer, Liam M. Crowley, University of Oxford and Wytham Woods Genome Acquisition Lab, Darwin Tree of Life Barcoding Collective, Wellcome Sanger Institute Tree of Life Management, Samples and Laboratory team, Wellcome Sanger Institute Scientific Operations: Sequencing Operations, Wellcome Sanger Institute Tree of Life Core Informatics team, Tree of Life Core Informatics collective, Darwin Tree of Life Consortium

**Affiliations:** 1 Independent researcher, Ventnor, Isle of Wight, England, UK; 2 University of Oxford, Oxford, Oxfordshire, England, UK

**Keywords:** Amara aenea, Common Sun Beetle, genome sequence, chromosomal, Coleoptera

## Abstract

We present a genome assembly from an individual male *Amara aenea* (Common Sun Beetle; Arthropoda; Insecta; Coleoptera; Carabidae). The assembly contains two haplotypes with total lengths of 880.37 megabases and 779.72 megabases. Most of the haplotype 1 assembly (91.85%) was scaffolded into 18 chromosomal pseudomolecules, including the X sex chromosome. Haplotype 2 was assembled to scaffold level. The mitochondrial genome has also been assembled, with a length of 16.33 kilobases. This assembly was generated as part of the Darwin Tree of Life project, which produces genomes for eukaryotic species found in Britain and Ireland.

## Species taxonomy

Eukaryota; Opisthokonta; Metazoa; Eumetazoa; Bilateria; Protostomia; Ecdysozoa; Panarthropoda; Arthropoda; Mandibulata; Pancrustacea; Altocrustacea; Allotriocarida; Hexapoda; Insecta; Dicondylia; Pterygota; Neoptera; Eumetabola; Endopterygota; Aparaglossata; Neuropteroidea; Coleoptera; Adephaga; Caraboidea; Carabidae; Harpalinae; Zabrini; *Amara*; *Amara aenea* (De Geer, 1774) (NCBI:txid585988)

## Background

*Amara aenea* (De Geer, 1774), the common sun beetle, is a ground beetle in the family Carabidae. Adults are about 6–9 mm long and usually have a shiny brassy or green metallic upper surface, with dark legs, paler tibiae and three pale basal antennal segments. The fine elytral striae and sharp, linear pronotal foveae help distinguish it from similar *Amara* species ( Brock, 2021; Lindroth, 1974).

*Amara aenea* is associated with dry, open and sunny ground, including grassland, agricultural land, gardens and sandy sites with sparse vegetation ( Brock, 2021; Lindroth, 1974). It is common in England and Wales and more local, though widely distributed, in Scotland and Ireland. Its wider range extends through most of Europe, but not the far north ( Luff, 1998). Adults are diurnal and fully winged, and the species breeds in spring ( Luff, 1998).

The genus *Amara* is largely associated with open habitats. Unlike many ground beetles, adult *Amara* feed substantially on seeds and other plant material ( Lindroth, 1974).

We present a chromosome-level genome sequence for *Amara aenea*, produced using the Tree of Life pipeline. The sequence is based on a specimen collected from Radley Large Wood, Oxfordshire, UK (Figure 1). This assembly was generated as part of the Darwin Tree of Life Project, which aims to generate high-quality reference genomes for all named eukaryotic species in Britain and Ireland to support research, conservation, and the sustainable use of biodiversity ( Darwin Tree of Life Project Consortium, 2022).

Although numerous genomes have been sequenced for the family Carabidae, this assembly provides the first chromosome-level genome assembly for *Amara aenea*, enabling comparative analyses (data obtained via NCBI datasets, O’Leary *et al.*, 2024). This is currently the only genome assembly available for this species. This assembly is the RefSeq reference assembly for this species.

**Figure 1.**
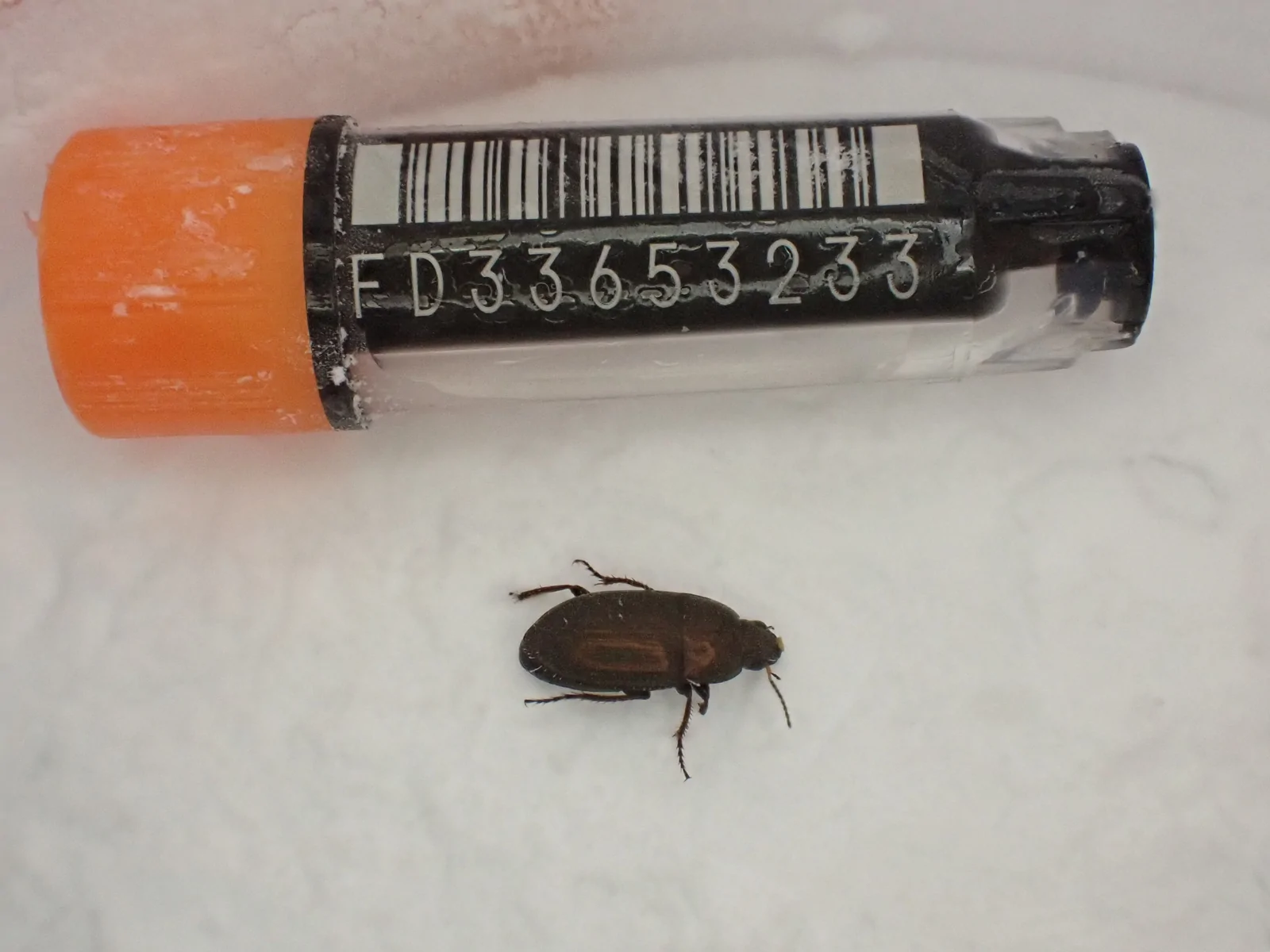
The *A. aenea* specimen (Ox003478, icAmaAene1) used for genome sequencing.

## Methods

### Sample acquisition and DNA barcoding

The specimen used for genome sequencing was an adult male *Amara aenea* (specimen ID Ox003478, ToLID icAmaAene1; Figure 1). The same specimen was also used for Hi-C sequencing. It was collected from Radley Large Wood, Oxfordshire, UK (latitude 51.705, longitude −1.25) on 2023-05-11 by Mark Telfer and Liam Crowley and identified by Mark Telfer.

The initial identification was verified by an additional DNA barcoding process according to the framework developed by Twyford *et al.*. (2024). A small sample was dissected from the specimen and stored in ethanol, while the remaining parts were shipped on dry ice to the Wellcome Sanger Institute (WSI) (see the protocol). The tissue was lysed, the COI marker region was amplified by PCR, and amplicons were sequenced and compared to the BOLD database, confirming the species identification ( Crowley *et al.* , 2023). Following whole-genome sequencing, the relevant DNA barcode region was also used alongside the initial barcoding data for sample tracking at the WSI ( Twyford *et al.* , 2024). The standard operating procedures for Darwin Tree of Life barcoding are available on protocols.io.

### Nucleic acid extraction

High molecular weight (HMW) DNA extraction protocols developed by the Tree of Life Core Laboratory at the Wellcome Sanger Institute (WSI) are available on protocols.io ( Howard *et al.*, 2025). Tissue from the icAmaAene1 sample was weighed and triaged to select the appropriate extraction workflow.

HMW DNA was prepared for one PacBio HiFi library: DTOL14798543. Tissue from the whole organism was homogenised by PowerMash using a PowerMasher II tissue disruptor. HMW DNA was extracted using the Automated MagAttract v2 protocol. DNA was sheared into an average fragment size of 12–20 kb following the Megaruptor®3 for LI PacBio protocol, and then purified by Automated SPRI.

The concentration of the sheared and purified DNA was assessed with a NanoDrop spectrophotometer and a Qubit Fluorometer using the Qubit dsDNA High Sensitivity Assay kit. Fragment size distribution was evaluated by running the sample on the Femto Pulse system. The final post-shearing DNA measurements included a Qubit concentration of 23.8 ng/μL, an average fragment size of 13.9 kb and a yield of 2,600.00 ng.

### PacBio HiFi library preparation and sequencing

Library DTOL14798543 was prepared with the SMRTbell Prep Kit 3.0 (Pacific Biosciences) according to the manufacturer’s instructions. The kit includes reagents for end repair/A-tailing, adapter ligation, post-ligation SMRTbell bead clean-up, and nuclease treatment. Size selection and clean-up were performed using diluted AMPure PB beads (Pacific Biosciences). DNA concentration was quantified with a Qubit Fluorometer v4.0 (Thermo Fisher Scientific) and the Qubit 1X dsDNA HS assay kit. Final library fragment size was assessed with the Agilent Femto Pulse Automated Pulsed Field CE Instrument (Agilent Technologies) using the gDNA 55 kb BAC analysis kit.

The library was sequenced on a Revio instrument (Pacific Biosciences, California, USA). Prepared libraries with a molarity greater than 2 nM were normalised to 2 nM, and 15 μL was used to make complexes. For libraries with a molarity below 2 nM, the available 10 μL library volume was used without normalisation. Primers were annealed and polymerases were bound to generate circularised complexes following the manufacturer’s instructions. Complexes were purified using 1.2× SMRTbell beads, diluted to a loading concentration of 200–300 pM, and spiked with a Revio sequencing internal control. Sequencing used Revio 25M SMRT cells. SMRT Link software was used to configure and monitor the run and to perform primary and secondary data analysis.

### Hi-C

***Sample preparation and crosslinking***

Hi-C data were generated from the icAmaAene1 whole organism sample using the Arima-HiC v2 kit (Arima Genomics). Following the manufacturer’s instructions, tissue was fixed and DNA crosslinked using TC buffer to a final formaldehyde concentration of 2%. The tissue was homogenised using the Diagnocine Power Masher-II. Crosslinked DNA was digested with a restriction enzyme master mix, biotinylated, and ligated. Clean-up was performed with SPRISelect beads before library preparation. DNA concentration was measured with the Qubit Fluorometer (Thermo Fisher Scientific) and Qubit HS Assay Kit. The biotinylation percentage was estimated using the Arima-HiC v2 QC beads.

***Hi-C library preparation and sequencing***

Biotinylated DNA constructs were fragmented using a Covaris E220 sonicator and size-selected to 400–600 bp using SPRISelect beads. DNA was enriched with Arima-HiC v2 kit Enrichment beads. End repair, A-tailing, and adapter ligation were carried out with the NEBNext Ultra II DNA Library Prep Kit (New England Biolabs), following a modified protocol where library preparation occurs while DNA remains bound to the Enrichment beads. Libraries were amplified with KAPA HiFi HotStart mix and a custom Unique Dual Index (UDI) barcode set (Integrated DNA Technologies). Depending on sample concentration and biotinylation percentage determined at the crosslinking stage, libraries were amplified with 10–16 PCR cycles. Post-PCR clean-up was performed with SPRISelect beads. Libraries were quantified using the AccuClear Ultra High Sensitivity dsDNA Standards Assay Kit (Biotium) and a FLUOstar Omega plate reader (BMG Labtech).

Prior to sequencing, libraries were normalised to 10 ng/μL. Normalised libraries were quantified again to create equimolar and/or weighted 2.8 nM pools. Pool concentrations were checked using the Agilent 4200 TapeStation (Agilent) with High Sensitivity D500 reagents before sequencing. Libraries were sequenced on the Illumina NovaSeq X, generating paired-end 150-bp reads.

### Genome assembly

Prior to assembly of the PacBio HiFi reads, a database of *k*-mer counts ( *k* = 31) was generated from the filtered reads using FastK. The *k*-mer frequency distribution was analysed with GenomeScope (version 2.1.0) ( Ranallo-Benavidez *et al.*, 2020), providing fitted point estimates of haploid genome size, heterozygosity and repeat content.

The HiFi reads were assembled using Hifiasm in Hi-C phasing mode ( Cheng *et al.*, 2021, 2022), producing two haplotypes. Hi-C reads ( Rao *et al.* , 2014) were mapped to contigs using bwa-mem2 ( Vasimuddin *et al.*, 2019). Contigs were further scaffolded with Hi-C data in YaHS ( Zhou *et al.*, 2023), using the --break option for handling potential misassemblies. The scaffolded assemblies were evaluated using Gfastats ( Formenti *et al.* , 2022), BUSCO ( Tegenfeldt *et al.*, 2025) and MerquryFK ( Rhie *et al.*, 2020).

The mitochondrial genome was assembled using MitoHiFi ( Uliano-Silva *et al.* , 2023). The MitoHiFi reference was the mitochondrial genome of *Amara ovata* (NC_067049.1).

### Assembly curation

The assembly was decontaminated using the Assembly Screen for Cobionts and Contaminants ( ASCC) pipeline. The flat files and maps for curation were generated with TreeVal. Manual curation was conducted primarily in PretextView and HiGlass ( Kerpedjiev *et al.* , 2018). Scaffolds were visually inspected and corrected as described by Howe *et al*. (2021). The two haplotype assemblies were combined for curation. Manual corrections included 12 breaks and 159 joins. This reduced the scaffold count by 9.1%, increased the scaffold N50 by 139.8%, and increased the total assembly length by 6.2%. The curation process is described at sanger-tol/curation-resources. A Hi-C contact map of the haplotype 1 assembly was generated with PretextSnapshot.

### Assembly quality assessment

The MerquryFK tool ( Rhie *et al.*, 2020) was run in a Singularity container ( Kurtzer *et al.*, 2017) to evaluate *k*-mer completeness and assembly quality for both haplotypes using the *k*-mer database ( *k* = 31) computed prior to genome assembly. The analysis outputs included assembly QV scores and completeness statistics.

The haplotype 1 assembly was assessed using BlobToolKit ( Challis *et al.*, 2020). PacBio reads were mapped to the assembly to derive sequence coverage, and BUSCO ( Tegenfeldt *et al.*, 2025) was used to assess gene-set completeness. DIAMOND BLASTp searches of extracted BUSCO proteins and DIAMOND BLASTx searches of assembly sequences against UniProt Reference Proteomes ( Buchfink *et al.*, 2021), followed by BLASTn searches of sequences without DIAMOND BLASTx hits against the NCBI nt database ( Altschul *et al.*, 1990), supported taxonomic assignment. Coverage, sequence composition, taxonomic assignment and BUSCO results were integrated into a BlobDir for interactive inspection and visualisation. The snail and blob plots were rendered locally from this hosted BlobDir using BlobTk v0.8.3. The scaffold-length radial scale function for the snail plot was set to linear ( Challis & Blaxter, 2026). The blob plot used circular markers with area proportional to scaffold length ( Challis *et al.*, 2020).

Ancestral linkage groups were painted along the chromosome-level scaffolds of the haplotype 1 assembly. Complete BUSCO genes (single-copy and duplicated) from the coleoptera_odb12 lineage dataset were assigned to the 8 Coleoptera ancestral linkage groups (C1–C7 and CX) described by Maulana *et al.*. (2026) and plotted along chromosomes drawn to scale.

## Genome sequence report

### Sequence data

PacBio sequencing of the *A. aenea* specimen generated 99.26 Gb (gigabases) from 9.11 million reads. These reads were used to assemble the genome. GenomeScope analysis using a *p* = 2 model gave a *k*-mer-based haploid genome size estimate of 765.41 Mb, with heterozygosity of 2.72% and repeat content of 46.74%; the full-model fit was 93.89% (Figure 2). The GenomeScope estimates guided expectations for the assembly. The NCBI assembly record reports 126× genome coverage. Hi-C sequencing produced 112.81 Gb from 373.55 million read pairs, which were used to scaffold the haplotype 1 assembly. Table 1 summarises the specimen and sequencing details.

**Figure 2.**
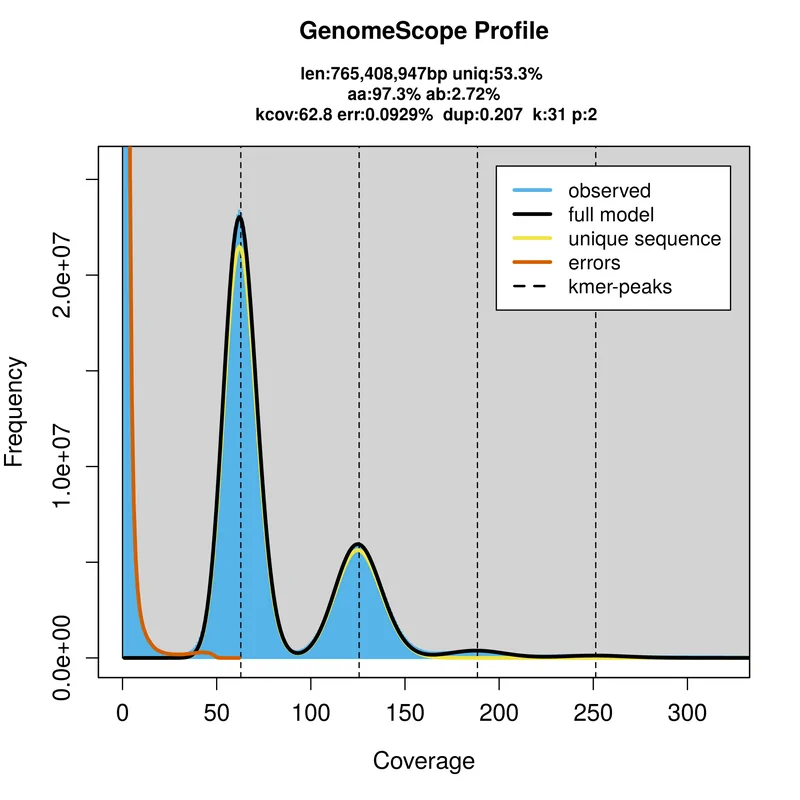
GenomeScope model fitted to the *k*-mer frequency distribution. The plot shows the observed and modelled *k*-mer spectra used to estimate haploid genome size, heterozygosity and repeat content from unassembled sequencing reads.

**Table 1.** Specimen and sequencing data for *A. aenea* (BioProject PRJEB78342)

**Platform**	**PacBio HiFi**	**Hi-C**
**ToLID**	icAmaAene1	icAmaAene1
**Specimen ID**	Ox003478	Ox003478
**BioSample (source individual)**	SAMEA114644544	SAMEA114644544
**BioSample (tissue)**	SAMEA114645157	SAMEA114645157
**Tissue**	whole organism	whole organism
**Instrument**	Revio	Illumina NovaSeq X
**Library ID & run accession(s)**	DTOL14798543: ERR13420783	SQPP-58013-Q:B3: ERR13389768
**Read count total**	9.11 million reads	373.55 million read pairs
**Base count total**	99.26 Gb	112.81 Gb

### Assembly statistics

The genome was assembled into two haplotypes using Hi-C phasing. The haplotype 1 assembly was curated to chromosome level, while haplotype 2 was assembled to scaffold level. The haplotype 1 assembly, which is proposed as the reference assembly for this species, has a total length of 880.37 Mb in 884 scaffolds (excluding organelle sequences), with 321 gaps, and its scaffold N50 is 46.48 Mb (Table 2). The scaffold auN for the haplotype 1 assembly is 42.0 Mb.

**Table 2.** Genome assembly statistics for *A. aenea*

**Genome assembly**	**Haplotype 1**	**Haplotype 2**
**Assembly name**	icAmaAene1.hap1.1	icAmaAene1.hap2.1
**Assembly accession**	GCA_973350105.1	GCA_973897575.1
**Assembly level**	chromosome	scaffold
**Span (Mb)**	880.37	779.72
**Number of chromosomes**	18	-
**Number of contigs**	1,205	826
**Contig N50 (Mb)**	6.36	6.2
**Number of scaffolds (excluding organelle sequences)**	884	602
**Scaffold N50 (Mb)**	46.48	44.13
**Scaffold auN (Mb)**	42.0	37.9
**Longest scaffold length (Mb)**	56.43	50.03
**Sex chromosomes**	X	-
**Organelles**	Mitochondrial genome: 16.33 kb	-

Most of the haplotype 1 assembly sequence (91.85%) was assigned to 18 chromosome-level scaffolds, representing 17 autosomes and the X sex chromosome. These chromosome-level scaffolds, confirmed by Hi-C data, are named according to size (Figure 3; Table 3). Chromosome X was identified by copy number in the diploid assembly. There was no identifiable Y sequence in the assembly however it may be present in the unplaced scaffolds or the species may be XO.

The mitochondrial genome was also assembled (length 16.33 kb, OZ313299.1). This sequence is included as a contig in the multifasta file of the haplotype 1 genome submission and as a standalone record.

**Figure 3.**
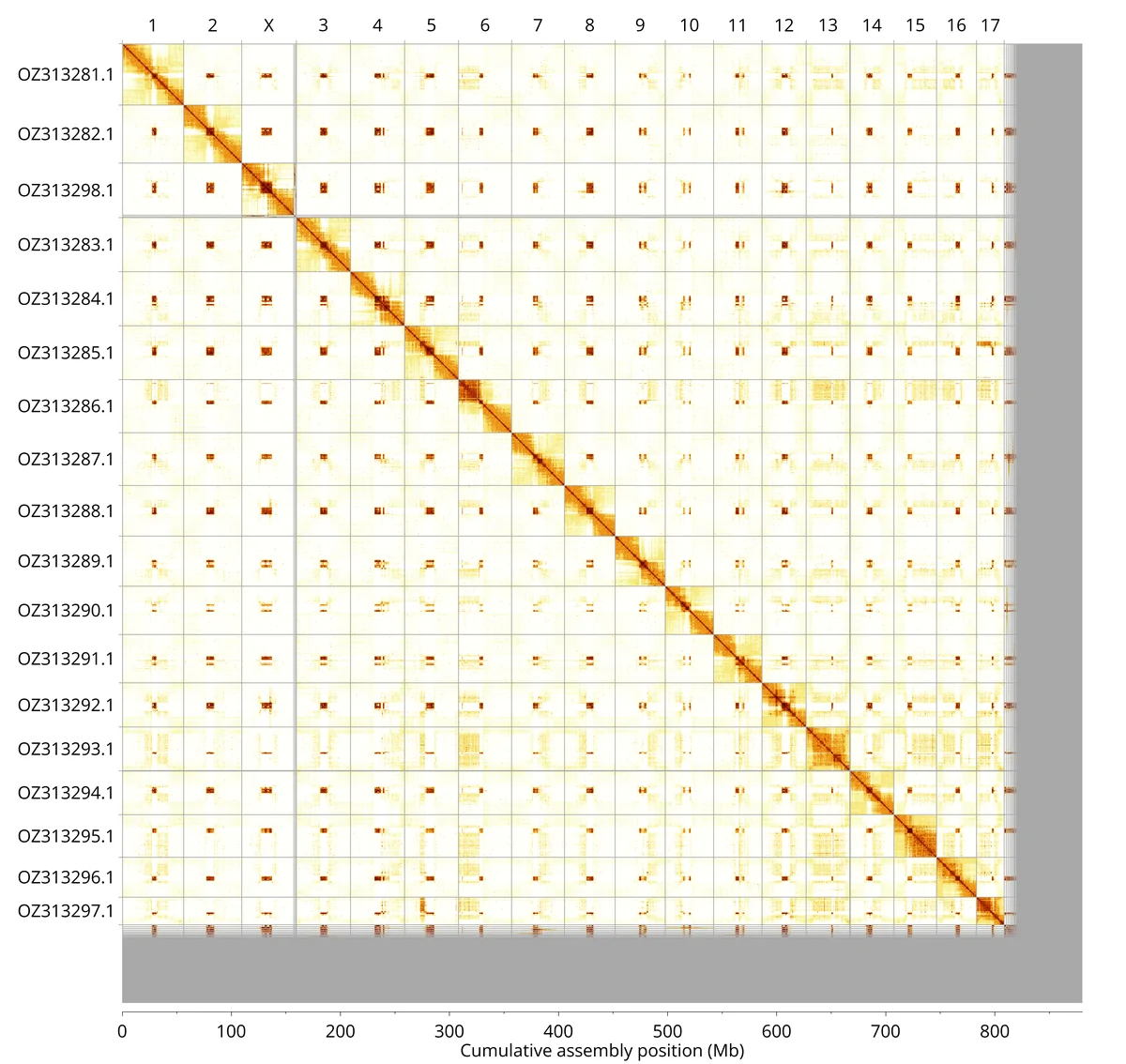
Hi-C contact map of the *A. aenea* haplotype 1 assembly. Assembled chromosomes are shown in order of size and labelled along the axes, with a megabase scale shown below. The plot was generated using PretextSnapshot.

**Table 3.** Chromosomal pseudomolecules in the haplotype 1 genome assembly of *A. aenea* icAmaAene1

**INSDC accession**	**Molecule**	**Length (Mb)**	**GC%**
OZ313281.1	1	56.43	31
OZ313282.1	2	53.28	30
OZ313283.1	3	49.64	30
OZ313284.1	4	49.48	30
OZ313285.1	5	49.44	30
OZ313286.1	6	48.61	31
OZ313287.1	7	48.45	30
OZ313288.1	8	46.48	30
OZ313289.1	9	45.97	30.50
OZ313290.1	10	44.50	29.50
OZ313291.1	11	44.27	30.50
OZ313292.1	12	40.60	30.50
OZ313293.1	13	40.44	31.50
OZ313294.1	14	39.84	30.50
OZ313295.1	15	39.15	31
OZ313296.1	16	36.53	31
OZ313297.1	17	25.44	31
OZ313298.1	X	50.11	30

Chromosome painting shows the distribution of BUSCO genes assigned to Coleoptera ancestral linkage groups across the chromosome-level scaffolds of the haplotype 1 assembly (Figure 4).

**Figure 4.**
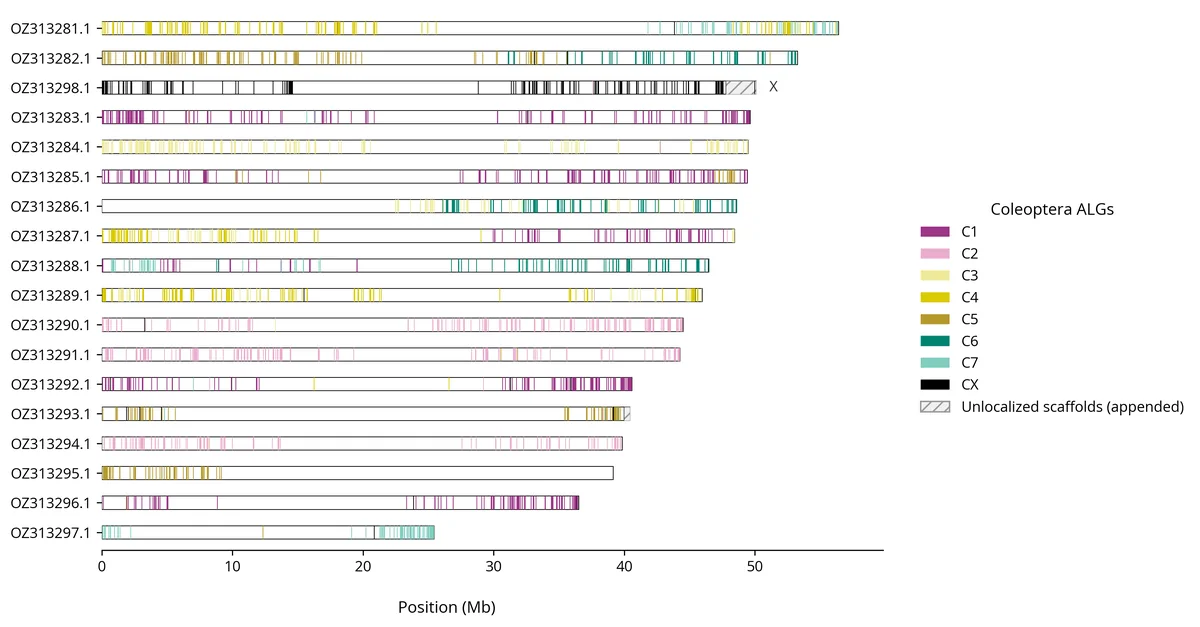
Coleoptera ancestral linkage groups painting of the haplotype 1 assembly of *A. aenea*. Chromosomes are drawn to scale, with coloured bars marking the positions of complete coleoptera_odb12 BUSCO genes. Each BUSCO gene is coloured by its assignment to one of the 8 Coleoptera ALGs (C1–C7 and CX).

### Assembly quality metrics

For the haplotype 1 assembly, the estimated QV is 58.9, and for the haplotype 2 assembly, 59.5. When the two haplotypes are combined, the assembly achieves an estimated QV of 59.1. The *k*-mer completeness is 67.43% for the haplotype 1 assembly, 62.61% for the haplotype 2 assembly, and 99.51% for the combined haplotypes (Figure 5).

**Figure 5.**
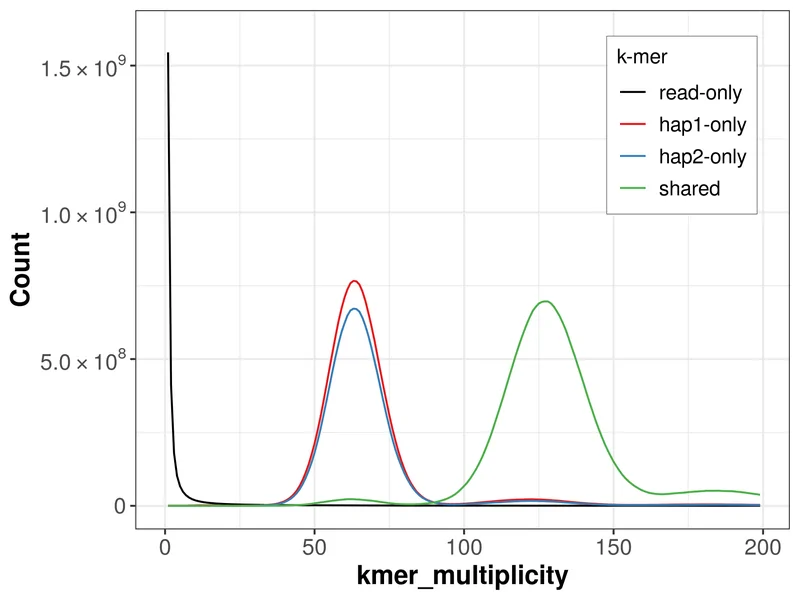
Evaluation of *k*-mer completeness using MerquryFK. This plot illustrates the recovery of *k*‐mers from the original read data in the haplotype 1 assembly, the haplotype 2 assembly and the combined haplotypes. The horizontal axis represents *k*‐mer multiplicity, and the vertical axis shows the number of *k*‐mers. The black curve represents *k*‐mers that appear in the reads but are not assembled. The green curve corresponds to *k*‐mers shared by both haplotypes, and the red and blue curves show *k*‐mers found only in one of the haplotypes.

BUSCO analysis using the endopterygota_odb10 reference set ( *n* = 2,124) identified 99.8% of the expected gene set (single = 98.3%, duplicated = 1.5%) in the haplotype 1 assembly. For the haplotype 2 assembly, BUSCO v.6.0.0 analysis identified 94.3% of the expected gene set (single = 93.1%, duplicated = 1.1%).

The snail plot in Figure 6 provides a summary of assembly contiguity, cumulative scaffold count, sequence composition and BUSCO completeness for the haplotype 1 assembly ( Challis & Blaxter, 2026). The relative sizes of the longest scaffold, scaffold N50 and N90, together with the dark-grey scaffold-length distribution, show how much of the assembly is contained in long scaffolds. The outer composition track shows variation in GC, AT and N content, while the central track shows the cumulative scaffold count and the BUSCO inset summarises gene-set completeness.

The blob plot in Figure 7 displays scaffolds from the haplotype 1 assembly by GC proportion and base coverage. Marker area is proportional to scaffold length, and colour indicates taxonomic assignment ( Challis *et al.*, 2020).

**Figure 6.**
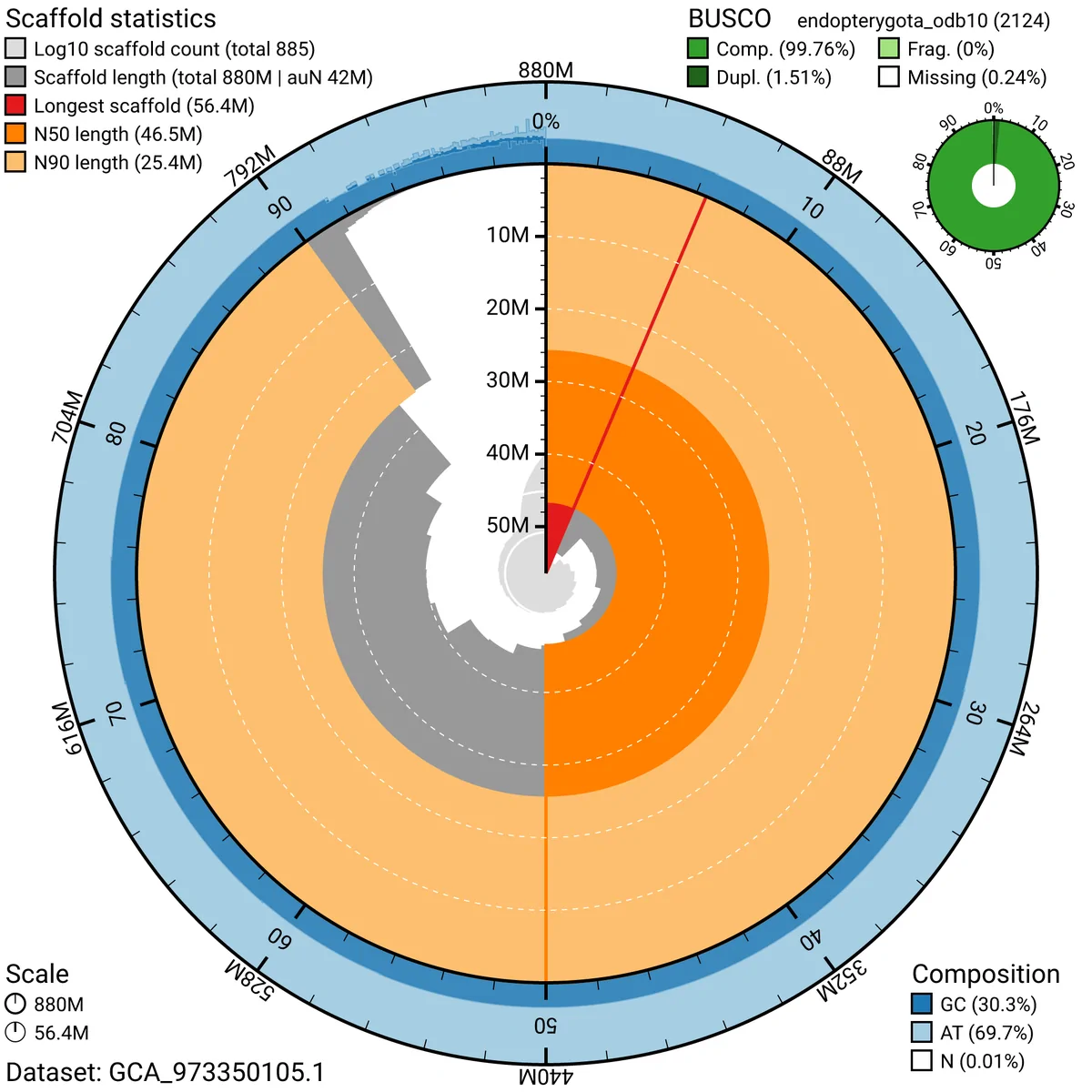
Assembly metrics for icAmaAene1.hap1.1, the haplotype 1 assembly. The BlobToolKit snail plot provides an overview of assembly metrics and BUSCO gene completeness. The circumference represents the length of the haplotype 1 assembly, and the main plot is divided into 1,000 bins around the circumference. The outermost blue tracks display the distribution of GC, AT, and N percentages across the bins. Scaffolds are arranged clockwise from longest to shortest and are depicted in dark grey. Scaffold length is shown on a linear radial scale, scaled to the longest scaffold. Red shading indicates the longest scaffold, and the deeper orange and pale orange shading represent the N50 and N90 lengths. The summary statistics include scaffold auN, the area under the scaffold Nx curve. The light grey spiral at the centre shows the cumulative scaffold count on a logarithmic scale. A summary of complete, fragmented, duplicated, and missing BUSCO genes in the endopterygota_odb10 set is presented at the top right. The assembly can be explored interactively in the BlobToolKit viewer.

**Figure 7.**
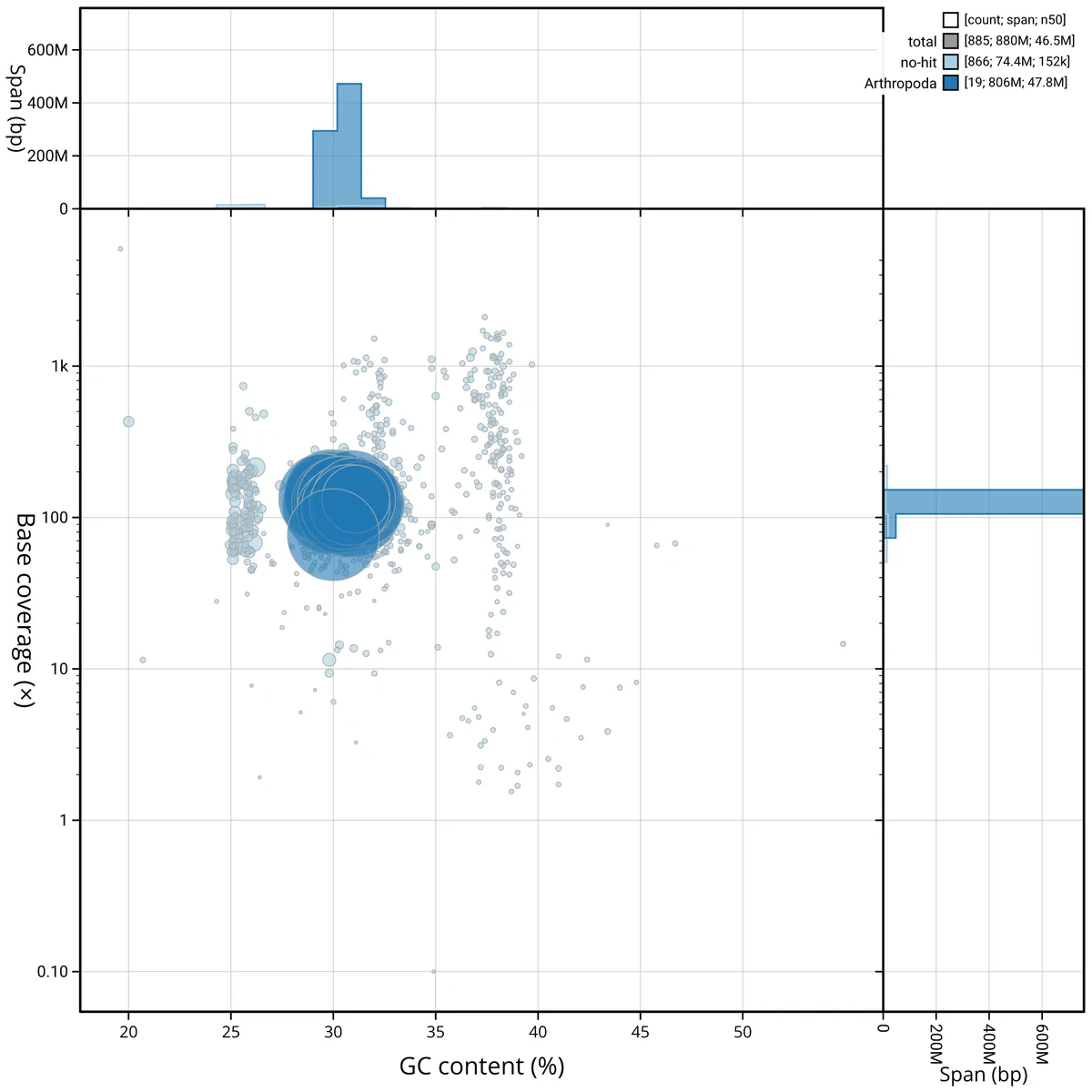
BlobToolKit blob plot for icAmaAene1.hap1.1, the haplotype 1 assembly. The plot shows base coverage (vertical axis) and GC content (horizontal axis). Circles represent scaffolds; marker area is proportional to scaffold length, and colour indicates taxonomic assignment. The histograms along the axes display the total length of sequences distributed across different levels of coverage and GC content. The assembly can be explored interactively in the BlobToolKit viewer.

Table 4 presents assembly quality metrics alongside the Earth BioGenome Project (EBP) benchmarks described by Howe *et al.*. (2026). The three-part EBP metric, calculated for the haplotype 1 assembly, is **6.C.Q58**.

**Table 4.** Earth BioGenome Project summary metrics for the *A. aenea* assembly

**Measure**	**Value**	***Benchmark***
EBP summary (haplotype 1)	6.C.Q58	*6.C.Q40*
Contig N50 length	6.36 Mb	*> 1 Mb*
Scaffold N50 length	46.48 Mb	*= chromosome N50*
Consensus quality (QV)	Haplotype 1: 58.9; haplotype 2: 59.5; combined: 59.1	*≥ 40*
*k*-mer completeness	Haplotype 1: 67.43%; Haplotype 2: 62.61%; combined: 99.51%	*> 90% (combined assembly)*
BUSCO	Haplotype 1: C:99.8% [S:98.3%, D:1.5%], F:0.0%, M:0.2%, n:2,124; Haplotype 2: C:94.3% [S:93.1%, D:1.1%], F:0.2%, M:5.5%, n:2,124	*S > 90%*
Percentage of assembly assigned to chromosomes	91.85%	*≥ 90%*

**Notes:** The EBP summary uses log10(Contig N50); chromosome-level (C) or log10(Scaffold N50); Q (Merqury QV). For *k*-mer completeness, the benchmark is assessed using the combined assembly value. BUSCO v6.0.0 using the endopterygota_odb10 lineage dataset: C=complete; S=single-copy; D=duplicated; F=fragmented; M=missing; n=orthologues.

### Data availability

European Nucleotide Archive: Amara aenea. Accession number PRJEB78342; https://identifiers.org/ena.embl/PRJEB78342. The genome sequence is released openly for reuse. The *A. aenea* genome sequencing initiative is part of the Darwin Tree of Life Project ( PRJEB40665) and the Sanger Institute Tree of Life Programme ( PRJEB43745). All raw sequence data and the haplotype assemblies have been deposited in INSDC databases. The genome will be annotated using available RNA-Seq data and presented through the Ensembl pipeline at the European Bioinformatics Institute. Raw data and assembly accession identifiers are reported in Table 1 and Table 2.

Production code used in genome assembly at the WSI Tree of Life is available at https://github.com/sanger-tol. Table 5 lists software versions used in this study.

**Table 5.** Software versions and sources

**Software**	**Version**	**Source**
BLAST	2.14.0	https://ftp.ncbi.nlm.nih.gov/blast/executables/blast+/
BlobToolKit	4.4.6	https://github.com/genomehubs/blobtoolkit
BlobTk (hosted BlobDir)	0.5.1	https://github.com/genomehubs/blobtk
BlobTk (snail and blob plot renderer)	0.8.3	https://github.com/genomehubs/blobtk
BUSCO	6.0.0; 6.1.0	https://gitlab.com/ezlab/busco
busco-alg-painter	0.1.0	https://github.com/Karenvn/busco-alg-painter
bwa-mem2	2.2.1	https://github.com/bwa-mem2/bwa-mem2
DIAMOND	2.1.8	https://github.com/bbuchfink/diamond
fasta_windows	0.2.4	https://github.com/tolkit/fasta_windows
FastK	1.2	https://github.com/thegenemyers/FASTK
GenomeScope	2.1.0	https://github.com/tbenavi1/genomescope2.0
Gfastats	1.3.6	https://github.com/vgl-hub/gfastats
Hifiasm	0.19.8-r603	https://github.com/chhylp123/hifiasm
HiGlass	1.13.4	https://github.com/higlass/higlass
MerquryFK	1.1.0-c1	https://github.com/thegenemyers/MERQURY.FK
Minimap2	2.28-r1209	https://github.com/lh3/minimap2
MitoHiFi	3.2.2	https://github.com/marcelauliano/MitoHiFi
MultiQC	1.14; 1.17 and 1.18	https://github.com/MultiQC/MultiQC
Nextflow	24.10.4	https://github.com/nextflow-io/nextflow
PretextSnapshot	0.0.6	https://github.com/sanger-tol/PretextSnapshot
PretextView	1.0.3	https://github.com/sanger-tol/PretextView
samtools	1.21	https://github.com/samtools/samtools
sanger-tol/ascc	0.1.0	https://github.com/sanger-tol/ascc
sanger-tol/blobtoolkit	v0.9.0	https://github.com/sanger-tol/blobtoolkit
sanger-tol/curationpretext	1.4.2	https://github.com/sanger-tol/curationpretext
Seqtk	1.4-r122	https://github.com/lh3/seqtk
Singularity	3.9.0	https://github.com/sylabs/singularity
TreeVal	1.1.0	https://github.com/sanger-tol/treeval
YaHS	1.2a.2	https://github.com/c-zhou/yahs

### Author information

Contributors are listed at the following links:

- Members of the University of Oxford and Wytham Woods Genome Acquisition Lab
- Members of the Darwin Tree of Life Barcoding collective
- Members of the Wellcome Sanger Institute Tree of Life Management, Samples and Laboratory team
- Members of Wellcome Sanger Institute Scientific Operations – Sequencing Operations
- Members of the Wellcome Sanger Institute Tree of Life Core Informatics team
- Members of the Tree of Life Core Informatics collective
- Members of the Darwin Tree of Life Consortium

### Wellcome Sanger Institute – Legal and Governance

The materials that have contributed to this genome note have been supplied by a Darwin Tree of Life Partner. The submission of materials by a Darwin Tree of Life Partner is subject to the **‘Darwin Tree of Life Project Sampling Code of Practice’**, which can be found in full on the Darwin Tree of Life website. By agreeing with and signing up to the Sampling Code of Practice, the Darwin Tree of Life Partner agrees they will meet the legal and ethical requirements and standards set out within this document in respect of all samples acquired for, and supplied to, the Darwin Tree of Life Project. Further, the Wellcome Sanger Institute employs a process whereby due diligence is carried out proportionate to the nature of the materials themselves, and the circumstances under which they have been/are to be collected and provided for use. The purpose of this is to address and mitigate any potential legal and/or ethical implications of receipt and use of the materials as part of the research project, and to ensure that in doing so we align with best practice wherever possible. The overarching areas of consideration are:

- Ethical review of provenance and sourcing of the material
- Legality of collection, transfer and use (national and international)

Each transfer of samples is further undertaken according to a Research Collaboration Agreement or Material Transfer Agreement entered into by the Darwin Tree of Life Partner, Genome Research Limited (operating as the Wellcome Sanger Institute), and in some circumstances, other Darwin Tree of Life collaborators.
