# Supplementary material for: The genome sequence of the Common Sun Beetle, Amara aenea (De Geer, 1774) (Coleoptera: Carabidae)
Source: Wellcome Open Res. 2026 Oct 9;11:841. [Version 1] doi: 10.12688/wellcomeopenres.28085.1 (PMC13736065; doi:10.12688/wellcomeopenres.28085.1)
Supplement: Peer review history

# Reviewer report: Reviewer response for version 1

**DOI:** 10.21956/wellcomeopenres.30901.r174465

**Published:** 2026-11-04

**Authors:** Ian M Carr

**Affiliations:** 1 University of Leeds, Leeds, UK

The paper is a standard Tree of Life paper and so is clearly explained. The only issue is Figure 3: This appears to be a screen grab of a program interface. The data in the figure doesn't fill the program's interface and so there is a lot of dead space in the figure consisting of greyed-out areas. The images needs this dead space editing out.

Are the datasets clearly presented in a usable and accessible format, and the assembly and annotation available in an appropriate subject-specific repository?

Yes

Are sufficient details of the sequencing and extraction, software used, and materials provided to allow replication by others?

Yes

Are the rationale for sequencing the genome and the species significance clearly described?

Yes

Are the protocols appropriate and is the work technically sound?

Yes

Reviewer Expertise:

NGS data analysis

I confirm that I have read this submission and believe that I have an appropriate level of expertise to confirm that it is of an acceptable scientific standard.

# Reviewer report: Reviewer response for version 1

**Published:** 2026-10-09

**Authors:** Automated Benchmarking

**Affiliations:** 1 Wellcome Sanger Institute, Hinxton, UK

As this article is a Genome Note, Automated Benchmarking has been used to determine whether the reported assembly passes four defined checks established by the Earth BioGenome Project: the raw and assembled data are publicly available, the Earth BioGenome Project three-part metric is met, the k-mer completeness exceeds 90%, and the BUSCO score passes the stated threshold. For Amara aenea (icAmaAene1), all four checks passed. Data available: pass. ENA study PRJEB78342, raw-read accessions ERR13420783 and ERR13389768, and assembly accessions GCA_973350105.1 and GCA_973897575.1 are public. Earth BioGenome Project three-part metric: pass. Haplotype 1 is reported as 6.C.Q58, meeting the 6.C.Q40 reference standard. k-mer completeness: pass. The combined haplotype assemblies have 99.51% k-mer completeness, exceeding the Earth BioGenome Project target of 90%. BUSCO: pass. Haplotype 1, which is proposed as the reference assembly for this species, has 99.8% complete BUSCOs (98.3% single-copy and 1.5% duplicated). BUSCO software version: 6.0.0; lineage dataset: endopterygota_odb10. It meets the target of greater than 90% complete and single-copy BUSCOs. This decision uses values reported in the Genome Note and public accession records.

Reviewer Expertise:

NA

I confirm that I have read this submission and believe that I have an appropriate level of expertise to confirm that it is of an acceptable scientific standard.
